# Nutrient Patterns and Body Composition Parameters of Black South African Women

**DOI:** 10.3390/nu13010006

**Published:** 2020-12-22

**Authors:** Caroline B. T. Makura-Kankwende, Philippe J. Gradidge, Nigel J. Crowther, Shane A. Norris, Tinashe Chikowore

**Affiliations:** 1 SAMRC/Wits Developmental Pathways for Health Research Unit (DPHRU), Faculty of Health Sciences, University of the Witwatersrand and Johannesburg, Johannesburg 2198, South Africa; Shane.Norris@wits.ac.za (S.A.N.); tinashe.chikowore1@wits.ac.za (T.C.); 2 Centre for Exercise Science and Sports Medicine, Faculty of Health Sciences, University of the Witwatersrand, Johannesburg 2198, South Africa; philippe.gradidge@wits.ac.za; 3 Department of Chemical Pathology, National Health Laboratory Service, Faculty of Health Sciences, University of the Witwatersrand, Johannesburg 2192, South Africa; Nigel.Crowther@nhls.ac.za

**Keywords:** African women, nutrient patterns, obesity, South Africa

## Abstract

Obesity is more prevalent in black South African women than men. However, little is known about the nutrient patterns associated with body composition indices in black African women. Principle Component Analysis (PCA) was applied to 25 nutrients derived from quantified food frequency questionnaires (QFFQs) in 498 middle aged black South African women. Three nutrient patterns, the plant driven, animal driven and Vitamin C, sugar and potassium driven nutrient patterns, accounted for 59% of the variance of nutrient intake. Linear models of the body composition parameters as outcome variables indicated that a standard deviation increase in the animal driven nutrient pattern was significantly associated with increases in body mass index (BMI) (1.29 kg·m^−2^ (95% CI, 0.54–2.04; *p* = 0.001), subcutaneous adipose tissue (SAT) (26.30 cm^2^ (7.97–44.63); *p* = 0.005), visceral adipose tissue (VAT) (9.88 cm^2^ (5.13–14.63); *p* < 0.001), VAT/SAT ratio (0.01 (0.00–0.02); *p* = 0.018), whole body fat mass index (0.74 kg·m^−2^ (0.25–1.22); *p* = 0.003), and whole body lean mass index (0.53 kg·m^−2^ (0.23–0.83); *p* = 0.001). An increase in plant driven nutrient pattern was significantly associated with an increase in SAT of 20.45 cm^2^ (0.47–40.43); *p* = 0.045. This study demonstrates that animal driven nutrient pattern, characterised by the consumption of more animal protein and fat nutrients, similar to the western diet is associated with increased body fat and lean mass.

## 1. Introduction

The obesity pandemic remains a growing global concern, having a direct impact on non-communicable diseases (NCDs) such as hypertension, type 2 diabetes, and cardiovascular diseases [1]. The prevalence of obesity is increasing in low and middle income countries (LMICs) such as South Africa, in part due to the ongoing nutritional transition and rapid urbanisation [2,3]. In a study conducted in black South African women residing in Soweto, Johannesburg, the prevalence of obesity was found to be 67.8% and the prevalence of morbid obesity (BMI ≥ 40) was 16.8% [4]. Similarly, results from the South African National Health and Examination Survey (SANHANES-1) conducted in 2012 reported that 40.1% of women were obese and 25.0% were overweight. These rates were higher than those in males, who reported obesity and overweight prevalence rates of 11.6% and 19.6%, respectively [5]. It is therefore pivotal to understand the determinants of obesity and body composition parameters that increase NCD risk in black African women as they carry a larger proportion of this burden compared to men.

Obesity is thought to develop from the interaction of genetic, environmental, and modifiable lifestyle factors such as diet and physical activity [6,7]. South Africa has experienced a shift from a more active lifestyle to a sedentary lifestyle, associated with increased overweight and obesity rates [2]. The adoption of a more Western diet, which mainly consists of a diet high in total and saturated fats and sugars and low consumption of whole grains such as legumes and home grown vegetables, is becoming prevalent [2]. More nutrition studies are now being conducted to understand the association of diet and obesity among South Africans.

A number of studies have focused on single nutrients and foods in evaluating the association of diet with obesity [8,9,10,11,12]. However, since people eat a combination of different foods, it is important that studies analyse nutrient pattern rather than individual food types. Nutrient pattern analysis enables the effect of the whole diet on health as well as body composition to be evaluated [13]. In view that nutrients are universal, the nutrient patterns can be compared across varied ethnicities. Only one study has evaluated the association of nutrient patterns with obesity in a black South African population and this was performed in adolescents [14]. However, findings from this study might not be transferrable to adults as these two groups are likely to have different eating patterns [15,16]. Therefore, more studies assessing the nutrient patterns association with body composition are required in black South Africans, particularly women, who carry the largest burden of obesity. Therefore, our study aimed to evaluate the association of nutrient patterns with body composition parameters in middle aged black South African women.

## 2. Materials and Methods

### 2.1. Study Population and Setting

We adopted a cross sectional study design nested in the longitudinal Birth to Twenty plus (Bt20) study, which is based at the Developmental Pathways for Health Research Unit, Chris Hani Baragwanath Hospital in Soweto, Johannesburg. The Bt20 study commenced in 1990 and recruited neonates, their mothers, and caregivers, who are being followed up at set time points [17]. Data for the current study was collected among 498 black South African female caregivers from a Bt20 sub-study called Study of Women Entering and in Endocrine Transition (SWEET) study [14,15]. From the Bt20 original cohort, 902 women were invited to participate and 702 women were eligible for the SWEET study using the following exclusion criteria: <40 years and >60 years, pregnant, did not give consent, and ethnicity other than black African [18]. Within this group of 702 women, 498 women had nutrition related data and therefore were included in this study. 

In order to determine whether a sample size of 498 was sufficient to meet the aims of our study, we used the guidelines for factor analyses produced by Mundfrom et al. [19] and Kline [20]. According to the method of Mundfrom et al., a sample size calculation table is used, which considers the number of variables and factors used in the factor analysis. The current study used 25 variables (nutrients) and 3 factors (nutrient patterns) in a principal component analysis (PCA) and this would require a minimum sample size of 160, which is well below that of the actual sample size. The study was conducted according to the guidelines of the Declaration of Helsinki, and approved by the Human Research Ethics Committee (Medical) of University of the Witwatersrand (ethics number: M170718 (2017-09-26) and M110627 (2011-06-24).

### 2.2. Dietary Intake

Dietary intake data were collected using a standardised quantitative food frequency questionnaire (QFFQ), designed for the South African population [21]. The QFFQ consists of 214 food items that are representative of foods consumed by at least 3% of the population. This tool has been piloted and utilised extensively for the same population as described elsewhere [22,23,24]. Trained interviewers used high quality photographs of food items to assist with participant recall of food and beverage items consumed during the last seven days. The participants were asked to arrange the cards into three piles: foods eaten in the last seven days, occasionally, or never eaten and this was recorded. In the case of food items consumed in the last seven days, additional data on the frequency and quantity of consumption was collected. The participants were asked to estimate their habitual intake by selecting the most accurate representation of their portion size from either two dimensional actual size drawings of foods, household utensils, or three dimensional validated food models as described and validated by Steyn et al. [25]. These household measures were then converted to grams for the computation of average intake over a seven-day period. The QFFQ took approximately 40–60 min to administer and was captured and managed using the Research Electronic Data Capture (RedCap) software package, an electronic data capture tool hosted at University of the Witwatersrand [26].

Nutrient intake was calculated from the conversion of single food items using the nutrient analysis software FoodFinder3 developed and hosted by The South African Medical Research Council [27]. Over- and under-reporting of dietary energy intake was corrected by removing participants with total energy intake that was <3000 and >30,000 kJ as described by Vorster et al. [28]. Plausibility of energy intake reporting was evaluated using the ratio of energy intake (EI) to estimated energy requirement (EER) [29]. Estimated energy requirement (EER) was calculated using the Institute of Medicine (IOM) gender specific equations using the participant’s age, weight, height, and physical activity level (PA) as shown below [29]. A physical activity factor (PA) of 1.12 arising from the ratio of total energy expenditure and basal energy expenditure was considered for this study in view of the moderate physical activity that has been reported for this population.

EER = 354 − ((6.91·Age (yrs)) + PA·(9.36·Weight (kgs)) + 726·Height (m)).

Dietary energy intake reporting was classified using categories derived from the EI:EER ratio. Participants with EI:EER ratio less than 0.7 were classified as under-reporters, 0.7 to 1.42 plausible reporters, and greater than 1.42 as over-reporters [29]. In multivariable linear regression models, the plausible reporters were used as the reference group.

### 2.3. Body Composition 

#### 2.3.1. Simple Body Anthropometry 

Height and weight measurements were assessed while participants were barefoot and wearing light clothing. Height was measured using a wall mounted stadiometer (Holtain Ltd., Crosswell, UK) and reported to the nearest 0.1 cm and weight was measured using a digital scale (Dismedinc., Anjou, QC, Canada) and reported to the nearest 0.1 kg. These measurements were used to calculate BMI. A soft measuring tape was used to measure hip (greatest circumference at the hips) and waist (smallest girth above the umbilicus) circumferences to the nearest 0.5 cm. Trained technicians performed all body measurements. 

#### 2.3.2. Dual-Energy X-ray Absorptiometry 

Dual-energy X-ray Absorptiometry (DXA) scans were performed using a Hologic (Londonderry, NH, USA) DXA by a single trained technician. The scans were conducted to measure visceral adipose tissue and subcutaneous adipose tissue. Women removed clothing and all metal objects, wore surgical gowns for the procedure, and had whole body scans analyzed using whole body less head as many participants wore wigs or hair weaves that could not be removed and these hairpieces are similar in density to soft tissue and may have caused measurement artefact. During data collection, the DXA phantom was scanned each morning to examine the CV of the DXA machine and the CV was found to be less than 0.5% for all parameters. For this study, participants had subtotal (whole body less head) fat mass and lean mass measured as described in a previous study [30]. Fat mass was used to calculate whole body fat mass index (FMI) (fat mass (kg)/height (m^2^)) and lean mass was used to calculate whole body lean mass index (LMI) (whole body lean mass (kg)/height (m^2^)) [31]. (CVs for DXA parameters were <2% for total fat mass and 1% for fat-free soft tissue mass). 

### 2.4. Statistical Analysis 

Data were analysed using SPSS Statistics software version 25 (IBM, Chicago, IL, USA) and STATA version 15 (Statacorp., College Station, TX, USA) [32], [33]. Normality tests were conducted using Q-Q plots for continuous variables. These variables are described using median and interquartile ranges (IQR) for non-parametric data or mean ± standard deviation (SD) for normally distributed data.

Principal component analysis (PCA) was used to extract nutrient patterns from 25 nutrients derived from the QFFQ. Total available carbohydrates were divided into starch and total sugar. The nutrients were log transformed to remove the bias due to the different scales used to quantify the nutrients. The nutrient density approach of dividing the nutrient intake by the total energy intake was adopted to adjust for energy intake [34]. This was used to capture variability of nutrient intake independently from changes of energy intake. The PCA procedure was performed using the covariance matrix of the log transformed nutrients that were selected to capture the whole dietary intake as previously reported by Pisa et al. [14]. Varimax rotation was performed to improve the interpretability of the factor loadings [35]. Three principal components (PCs) representing three independent nutrient patterns were selected according to the percentage of total variance explained, their eigenvalues, and visual presentation on the scree-plot of eigenvalues (Supplementary Materials Figure S1).

Multivariable linear regression models were performed to assess the association of the selected body composition parameters as outcome variables and nutrient patterns as predictors while adjusting for dietary intake reporting (under-, plausable-, and over-reporting), employment status, age, physical activity, and smoking status. Statistical significance was accepted at a level of *p* < 0.05.

## 3. Results

### 3.1. Descriptive Characteristics 

Of the 498 women, 11.2% were underweight or normal weight (Table 1). The body composition indicators visceral adipose tissue (VAT), subcutaneous adipose tissue (SAT), FMI, LMI, gynoid fat, and hip and waist circumference were significantly higher in the overweight and obese group compared to the lean participants (*p* < 0.001 for all comparisons). The VAT/SAT ratio was not different between the groups (*p* = 0.171). Most of the women (65.4%) were classified as physically active and only 2.8% were active smokers. The proportion of energy intake from dietary carbohydrate and fat was similar across the groups (54.4% and 53.5% for carbohydrate intake and 31.3% and 30.1% for fat intake for lean and overweight/obese participants, respectively). However, the proportion of energy intake from protein was significantly different, with those obese or overweight consuming higher levels of protein compared to the lean participants (11.8% and 11.0%, respectively; *p* = 0.026).

### 3.2. Nutrient Patterns of Population

Table 2 shows that three nutrient patterns were extracted using PCA analysis, explaining 59% of the total variation of nutrients consumed by the study participants. The first nutrient pattern, the “*Plant driven nutrient pattern*” with higher factor loadings of plant protein, starch, and B vitamins, explained 25% of the variance; while the second (the “*Animal driven nutrient pattern*”), characterised by animal protein and saturated fat, and third (the “*Vitamin C, sugar and potassium driven nutrient pattern*”) accounted 23% and 11%, respectively. 

### 3.3. Factors Associated with Varying Body Composition Measurements

Statistically significant associations were found for the animal driven nutrient pattern with all body composition indicators (Table 3). A one standard deviation increase in the animal driven nutrient pattern resulted in a 1.19 kg·m^−2^ (*p* = 0.002) increase in BMI, 10.17 cm^2^ (*p* < 0.001) increase in VAT, 24.43 cm^2^ (*p* = 0.009) increase in SAT, 0.01 (*p* = 0.009) increase in VAT/SAT ratio, 0.69 kg·m^−2^ (*p* = 0.005) increase in FMI, and 0.48 kg·m^−2^ (*p* = 0.002) increase in LMI. There was a marginal association for plant driven nutrient pattern, with an increase in this pattern resulting in an increase in SAT of 20.45 cm^2^ (*p* = 0.045). Underweight participants were associated with over-reporting energy intake, while overweight individuals were noted to underreport. Notably, over-reporting energy intake, when compared to plausible energy intake, was associated with decreases of 3.51 kgm^−2^ in BMI, 17.61 cm^2^ in VAT, 76.15 cm^2^ in SAT, 1.95 kg·m^−2^ in FMI, and 1.47 kg·m^−2^ in LMI (*p* < 0.001 for all associations). Engagement in physical activity was associated with BMI, VAT, SAT, FMI, and LMI and inverse associations were reported at 1.51 kg·m^−2^, 9.21 cm^2^, 37.76 cm^2^, 0.91 kg·m^−2^, and 0.56 kg·m^−2^, respectively. Increasing age showed a direct statistically significant association with VAT (*p* < 0.001) and VAT/SAT ratio (*p* = 0.002). The plant protein and starch driven nutrient pattern was only associated with modest increases in SAT compared to the animal protein and fat driven pattern (Table 3).

## 4. Discussion

This study aimed to determine the nutrient patterns of middle aged black women in Soweto and their association with body composition measurements. Three nutrient patterns, namely, plant driven nutrient pattern, animal driven nutrient pattern and vitamin C, sugar and potassium driven nutrient pattern, were found to explain 59% of the variance in nutrient intake in the study population. The animal driven nutrient pattern was positively associated with BMI and DXA-derived body composition parameters. 

Three studies have previously characterised nutrient patterns of black Africans. Pisa et al. [14] and Visser et al. [36] studies were conducted in children and adolescents, while the study by Chikowore et al. [37] was done in adults. Pisa et al. found that the animal driven nutrient pattern comprising of animal protein, saturated fat, cholesterol, riboflavin, vitamin B12, retinol, vitamin D, and zinc were the most commonly consumed nutrient pattern, while in our study, this was the second most consumed pattern [14]; Visser et al. reported similar results to our study where the plant driven nutrient pattern comprising of protein, carbohydrate, iron, and vitamin B was the most consumed followed by the animal protein and saturated fat pattern [36]. Additionally, Chikowore et al. also reported similar findings to our study where the plant driven pattern comprising of magnesium, phosphorus, and plant protein was the most consumed pattern followed by fat and animal protein and starch driven nutrient patterns [37]. Regardless of the small differences in the naming of the patterns, Visser et al. and Chikowore et al. studies, conducted in both urban and rural areas, reported similar nutrient patterns to those that we found in our study [36,37]. This thereby suggests that these patterns are common in the black South African population. These nutrients are most likely derived from the consumption of staple foods such as maize meal, which are fortified with B vitamins [36,38] However, the differences in the variances of the animal driven nutrient pattern reported by Pisa et al. are expected, since the principals analysis approach used is data driven and thus, is expected to capture the unique patterns [14]. However, this pattern was also noted in our study and the high loadings of animal protein and saturated fat are suggestive of the shift towards western diets, which has been reported in this population group.

Very few studies have been conducted to assess the association between nutrient patterns and obesity. One such study conducted in American adults evaluated nutrient patterns and their relationship with general and central obesity [13]. Results from this study showed that the odds ratios of obesity increased across quartiles of the major nutrient pattern of saturated/mono-unsaturated fatty acids [13]. Relative to the first quartile, the fourth quartile was associated with odds ratio of 1.5 (95% CI, 1.3–1.7) for central obesity and odds ratio of 1.3 (95% CI: 1.1–1.5) for general obesity [13]. These findings are similar to those of the present study, which showed that the animal driven nutrient pattern is significantly associated with increases in BMI, VAT, SAT, VAT/SAT ratio, FMI, and LMI.

The plant driven nutrient pattern was correlated significantly with SAT, but not as strongly as the animal driven pattern. Although it has been demonstrated that a plant-based protein diet, through its anabolic properties, could be an effective strategy to enhance lean mass index, when eaten in excess, these foods may result in increasing SAT, hip, and waist circumference [39]. The increase in body parameters may be due to the fact that these plant-based foods may contain hidden sugars and refined carbohydrates, which contribute to weight gain [39].

The animal driven nutrient pattern, characterised with higher loadings for animal protein and fat driven nutrients, increased body composition parameters, potentially due to the storage of animal fat as triglycerides in adipose tissue [40,41]. However, protein is known to lead to increased energy expenditure and improved satiety through the release of hormones, which favours weight loss [42]. In fact, protein weight loss effects have been reported when it contributes approximately 20% or more of the energy intake [43]. This weight loss effect was less likely in our study, which comprised of protein intake at 11% of total energy intake. Furthermore, it must be noted that when energy demand is low, excess protein can be converted to ketone bodies or glucose (via gluconeogenesis) and contribute to a positive energy balance, which can result in increased weight [44]. Previous research done in both animals and humans demonstrated that a high fat diet promotes obesity [45]. This explains the association of the animal driven nutrient pattern with increases in body fat as measured by BMI. 

A strength of this study included using more accurate DXA body composition measurements when compared to simple measurements. Additionally, this study adjusted for energy intake misreporting in the linear regression models, which improved the accuracy of these models. Notably, overweight/obese subjects were noted to under report dietary energy intake as has been indicated in other studies [46,47]. The nutrient density method was implemented in order to adjust for total energy, thereby allowing for the independent association of the nutrients with body composition to be determined.

The study has some limitations. The QFFQ is known to have recall bias, including the potential for food measurement errors, resulting in inaccurate reporting of food consumption [48]. Recall bias was reduced by using actual food models to help with accurate recall of food quantities consumed. Similarly, physical activity was self-reported, however interviewers provided contextual examples of moderate and vigorous intensity activities during the interview to assist with recollection of recent activity. Accurate physical activity measurements using electronic activity monitors would ideally remove the potential for recall bias, however such tools are expensive and not feasible for large scale epidemiological studies [49].

## 5. Conclusions

This study showed that in this population, the animal driven nutrient pattern was significantly associated with increases in body fat and its distribution was indicated by the selected measures (BMI, VAT, SAT, VAT/SAT ratio, FMI, and LMI). This information is essential for interventions aimed at addressing obesity in black sub-Saharan African women. Decreased consumption of foods high in animal protein and fat will result in decreased body fat levels. However, larger studies are required to validate these findings.

## Figures and Tables

**Table 1:** Descriptive characteristics for the study population according to BMI.

	Overall	Underweight and Normal Weight (*N* = 56)	Overweight and Obese (*N* = 441)	*p* Value
Demographic Characteristics				
Age (years)	49 (45–53)	47 (44–52)	49 (45–53)	0.162
Current smoker *n* (%)	14 (2.8%)	2 (3.6%)	12 (2.7%)	0.063
Physically active *n* (%)	325 (65.4%)	41 (73.2%)	284 (64.4%)	0.191
Employed *n* (%)	295 (59.5%)	29 (52.7%)	266 (60.3%)	0.280
Body Composition Indicators				
Visceral Adipose Tissue (cm^2^)	99.7 (74.1–127.6)	42.3 (29.0–58.5)	103.1 (83.9–131.5)	<0.001
Subcutaneous Adipose Tissue (cm^2^)	455.8 (356.2–561.8)	215.8 (168.9–266.0)	482.0 (387.2–576.5)	<0.001
VAT/SAT ratio	0.2 (0.2–0.3)	0.2 (0.2–0.3)	0.2 (0.2–0.3)	0.171
Whole body fat mass index (FMI) (kg)	32.0 (25.6–39.3)	16.8 (12.8–19.4)	33.1 (28.4–40.7)	<0.001
Whole body lean mass index (LMI) (kg)	40.4 (35.8–44.8)	31.1 (28.7–34.6)	41.5 (37.2–45.4)	<0.001
Gynoid fat	58.6 (47.2–70.8)	32.7 (27.9–39.3)	61.3 (51.7–72.5)	<0.001
Hip circumference (cm)	117.5 (109.0–128.0)	97.0 (91.5–101.3)	119.0 (112.5–129.0)	<0.001
Waist circumference (cm)	99.5 (90.0–108.0)	77.0 (74.3–81.3)	101.5 (93.0–110.0)	<0.001
Dietary Information				
Total energy (kJ)	9759 (7628–13271)	10737 (8717–12659)	9614 (7523–13354)	0.291
% Carbohydrates	53.5(48.8–58.4)	54.4 (49.4–58.8)	53.5 (48.8–58.3)	0.598
% Protein	11.6 (10.3–13.3)	11.0 (9.7–12.4)	11.8 (10.4–13.4)	0.026
% Fat	30.2 (25.9–34.3)	31.3 (25.5–35.1)	30.1 (26.0–34.2)	0.756

**Table 2:** Factor loadings and explained variances for the nutrient patterns.

	Plant Protein Driven Nutrient Pattern	Animal Protein Driven Nutrient Pattern	Vitamin C, Sugar, and Potassium Driven Nutrient Pattern
Plant protein	**0.921**	0.121	−0.017
Animal protein	0.080	**0.834**	0.175
Saturated fat	0.284	**0.729**	0.136
Monounsaturated fat	0.352	**0.695**	0.062
Polyunsaturated fat	0.443	0.549	−0.059
Cholesterol	0.009	**0.776**	−0.003
Starch	**0.712**	0.046	−0.304
Total sugar	0.050	0.095	**0.721**
Dietary fibre	**0.686**	0.044	0.455
Calcium	−0.110	0.507	0.431
Iron	**0.785**	0.413	0.170
Magnesium	0.574	0.169	0.273
Phosphorus	0.156	0.560	0.171
Potassium	0.028	0.089	**0.688**
Zinc	**0.758**	0.471	0.117
Retinol	0.073	0.345	0.110
Beta carotene	0.033	−0.038	0.229
Thiamine	**0.861**	0.341	0.158
Riboflavin	0.415	**0.630**	0.314
Vitamin B6	**0.906**	0.199	−0.019
Folate	0.618	0.080	0.111
Vitamin B12	0.113	0.691	0.101
Vitamin C	0.193	0.063	**0.897**
Vitamin D	0.265	**0.837**	−0.124
Vitamin E	0.370	0.482	0.088
Explained variance %	24.678	22.945	10.884
Cumulative explained variance %	24.678	47.623	58.507

Bold factor loadings used to indicate factor loadings > ±0.60 used for naming the nutrient patterns.

**Table 3:** Multivariable linear regression models showing the association of body composition indicators (BMI, VAT, SAT, VAT/SAT, FMI, and LMI; outcome variables) with the three nutrient patterns (predictor variables) and possible confounding factors (energy intake reporting, physical activity, employment status, education, smoking, and alcohol intake).

		BMI		Visceral Adipose Tissue (VAT)		Subcutaneous Adipose Tissue (SAT)		VAT/SAT Ratio		Whole Body Fat Mass Index (FMI)		Whole Body Lean Mass Index (LMI)	
		Adjusted β (95% CI)	*p* Value	Adjusted β (95% CI)	*p* Value	Adjusted β (95% CI)	*p* Value	Adjusted β (95% CI)	*p* Value	Adjusted β (95% CI)	*p* Value	Adjusted β (95% CI)	*p* Value
Nutrient patterns	PCA1	0.65 (−0.17; 1.47)	0.118	2.06 (−3.12; 7.23)	0.434	20.45 (0.47; 40.43)	0.045	−0.01 (−0.01; 0.01)	0.309	0.46 (−0.07; 0.99)	0.088	0.20 (−0.12; 0.53)	0.220
	PCA2	1.29 (0.54; 2.04)	0.001	9.88 (5.13;14.63)	<0.001	26.30 (7.97; 44.63)	0.005	0.01 (0.00; 0.02)	0.018	0.74 (0.25; 1.22)	0.003	0.53 (0.23; 0.83)	0.001
	PCA3	0.30 (−0.31; 0.91)	0.328	−1.09 (−4.97; 2.80)	0.581	−5.05 (−20.04; 9.94)	0.508	0.00 (−0.01; 0.01)	0.567	0.21 (−0.19; 0.60)	0.300	0.08 (−0.16; 0.33)	0.506
EI-EER	Plausible	1		1		1		1		1		1	
	Under reporting	1.85 (−0.40; 4.10)	0.107	5.41 (−8.83; 19.64)	0.456	54.44 (−0.53; 106.41)	0.064	−0.00 (−0.04; 0.02)	0.695	1.49 (0.03; 2.95)	0.045	0.37 (−0.53; 1.26)	0.421
	Over reporting	−3.51 (−5.18; −1.85)	<0.001	−17.61 (−28.13; −7.09)	0.001	−76.15 (−116.77; −35.53)	<0.001	−0.00 (−0.02; 0.02)	0.972	−195 (−3.03; −0.87)	<0.001	−1.47 (−2.13; −0.80)	<0.001
Physical activity	Inactive	1		1		1		1		1		1	
	Physically Active	−1.51 (−2.76; −0.26)	0.018	−9.21 (−17.14; −1.28)	0.023	−37.76 (−68.37; −7.15)	0.016	−0.00 (−0.02; 0.01)	0.671	−0.91 (−1.73; −0.10)	0.027	−0.56 (−1.06; −0.06)	0.028
Employment	Unemployed	1		1		1		1		1		1	
status	Employed	1.41 (0.21; 2.62)	0.023	−2.10 (−9.79; 5.58)	0.590	26.07 (−3.59; 55.73)	0.085	−0.02 (−0.03; −0.01)	0.023	0.81 (0.03; 1.60)	0.043	0.55 (0.06; 1.03)	0.027
Education	Completed primary	1		1		1		1		1		1	
	Attended high school	−0.33 (−2.31; 1.64)	0.739	−2.77 (−15.27; 9.74)	0.643	12.35 (−35.92; 60.63)	0.615	−0.02 (−0.05; 0.04)	0.096	0.07 (−1.21; 1.35)	0.914	−0.39 (−1.17; 0.40)	0.336
	Completed high school	−2.13 (−4.28; 0.02)	0.052	−8.28 (−21.87; 5.31)	0.232	−23.53 (−76.04; 28.95)	0.379	−0.02 (−0.05; 0.001)	0.132	−1.00 (−2.39; 0.39)	0.157	−1.07 (−1.93; −0.22)	0.014
Cigarette use	Never smoked	1		1		1		1		1		1	
	Ex-smoker	−2.00 (−4.38; 0.36)	0.097	7.76 (−22.67; 7.16)	0.307	−31.28 (−88.88; 26.31)	0.286	0.00 (−0.03; 0.03)	0.909	−1.56 (−3.10; −0.31)	0.046	−0.40 (−1.34; 0.55)	0.406
	Current smoker	−0.24 (−4.00; 3.52)	0.899	−12.76 (−15.27; 9.74)	0.290	−23.53 (−76.01; 28.95)	0.379	−0.04 (−0.09; 0.01)	0.145	−0.32 (−2.76; 2.11)	0.794	0.09 (−1.41; 1.59)	0.905
Alcohol	No	1		1		1		1		1		1	
	Yes	−0.02 (−0.15; 0.10)	0.714	0.54 (−0.27; 1.36)	0.187	−0.83 (−3.96; 2.30)	0.603	0.00 (0.00; 0.00)	0.026	−0.01 (−0.09; 0.7)	0.849	−0.05 (−0.07; 0.04)	0.566
Age		0.03 (−0.08; 0.15)	0.569	1.68 (0.94; 2.42)	<0.001	2.61 (−0.27; 5.49)	0.075	0.00 (0.00; 0.00)	0.002	0.17 (−0.02; 0.35)	0.073	−0.03 (−0.07; 0.02)	0.205

PCA1 = Plant protein and starch driven nutrient pattern; PCA2 = Animal protein and fat driven nutrient pattern; PCA3 = Vitamin C, sugar, and potassium driven nutrient pattern.

## Data Availability

Please refer to suggested Data Availability Statements in section “MDPI Research Data Policies” at https://www.mdpi.com/ethics.

## Supplementary Materials

The following are available online at https://www.mdpi.com/2072-6643/13/1/6/s1, Figure S1: Scree plot of the nutrients and the extracted principal components.
