# Supplementary figures and images for: Nutrient Patterns and Body Composition Parameters of Black South African Women
Source: Nutrients. 2020 Dec 22;13(1):6. doi: 10.3390/nu13010006 (PMC7822018; doi:10.3390/nu13010006)

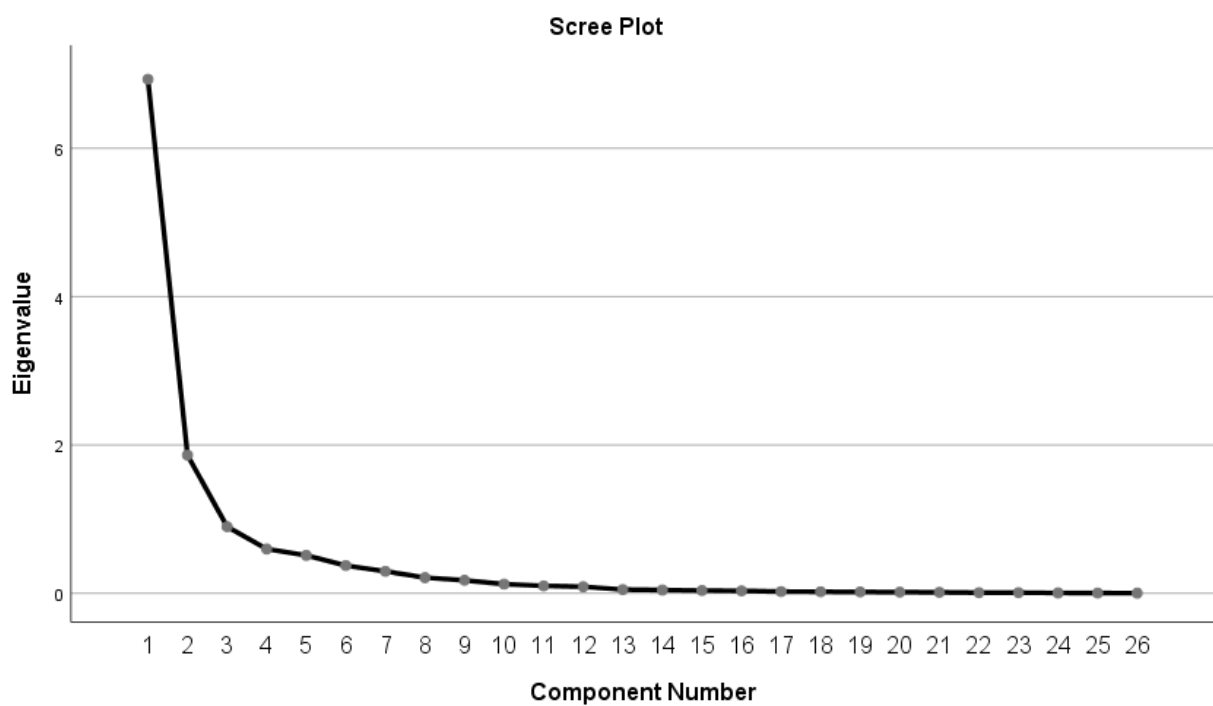

**Figure S1.** Scree plot of the nutrients and the extracted principal components.

Supplement: Supplementary file 1 [file nutrients-13-00006-s001.pdf]
